# Genomic Analysis of the ASMT Gene Family in *Solanum lycopersicum*

**DOI:** 10.3390/molecules22111984

**Published:** 2017-11-16

**Authors:** Weicheng Liu, Dake Zhao, Chunfang Zheng, Chen Chen, Xin Peng, Yuan Cheng, Hongjian Wan

**Affiliations:** 1 Zhengjiang Key Laboratory of Exploitation and Preservation of Coastal Bio-Resource, Zhejiang Mariculture Research Institute, Wenzhou 325000, China; lwch80@126.com (W.L.); diloma@sina.com (C.C.); pengxin_1128@163.com (X.P.); 2 Laboratory of Ecology and Evolutionary Biology, State Key Laboratory for Conservation and Utilization of Bio-Resources in Yunnan University, Kunming 650091, China; zhaodk2012@ynu.edu.cn; 3 Statekey Laboratory Breeding Base for Zhejiang Sustainable Pest and Disease Control, Institute of Vegetables, Zhejiang Academy of Agricultural Sciences, Hangzhou 310021, China; chenyuan1005@126.com

**Keywords:** Acetylserotonin methyltransferase (ASMT), identification and expression, biotic stresses, *Solanum lycopersicum*, melatonin biosynthesis

## Abstract

Acetylserotonin methyltransferase (ASMT) is the last enzyme of melatonin biosynthesis and may play a rate-limiting role in the melatonin production of plants. In this study, systematic analysis of the ASMT gene family in tomato (*Solanum lycopersicum* Mill) has been presented by the integration of the structural features, phylogenetic relationships, exon/intron configuration, and expression profile during growth and development, as well as biotic stresses. The results revealed that the tomato genome encoded a minimum of 14 members, containing three probable encoded pseudogenes. Chromosome mapping indicated that the family had probably expanded via tandem duplication events. Genome-wide RNA-seq and qRT-PCR based gene expression analysis revealed that almost half of the *SlASMT* genes were expressed in at least one of the experimental stages studied and also showed differential accumulation. Furthermore, the tandem duplicated *SlASMT* genes showed differential expression levels, which indicated probable functional divergence during the course of the evolution. Finally, this study also determined that some *SlASMT* genes were induced by multiple pathogens. The results suggested that these genes could be involved in tomato plant response to biotic stresses.

## 1. Introduction

Melatonin (*N*-acetyl-5-methoxytryptamine) has been identified as an indoleamine, with an unstable form. It widely exists in various tissues of higher plants, such as the roots, stems, leaves, flowers, fruit, and seeds [1,2,3,4,5,6]. Following the first report in 1995, it was determined that melatonin exists in almost every plant species [7,8]. Moreover, these studies have shown that melatonin plays an important role in regards to broad-spectrum antioxidants [9,10], biotic and abiotic stresses [11,12,13,14,15,16,17], seed dormancy, and the growth and development of plants [18,19,20]. Meanwhile, plants treated with exogenous melatonin in specific concentrations also have the ability to appropriately regulate resistance mechanisms and metabolic processes during growth and development stages [9,21,22,23].

In recent years, various studies have confirmed that melatonin synthesis in plants is involved with four different enzymes, including tryptophan decarboxylase (TrpDC), tryptamine 5-hydroxylase (T5H), serotonin *N*-acetyltransferase (SNAT), and acetylserotonin-*O*-methyltransferase (ASMT) [24,25]. However, when compared with that in animals, plant serotonin is purportedly synthesized first by the catalysis of tryptophan decarboxylase (TrpDC), followed by tryptamine 5-hydroxylase (T5H), rather than tryptophan 5-hydroxylase (Trp5H), and aromatic l-amino acid decarboxylase (AADC) as found in animals [24,25]. In plant species, previous results have shown that the over-expression of apple *MzASMT* improves melatonin production (2 to 4 times higher than that in the wild type)and enhances the drought tolerance of transgenic *Arabidopsis thaliana* plants [26]. In 2011, the first rice ASMT was cloned through the in vivo analysis of melatonin in recombinant *Escherichia coli*, and it was found that rice *ASMT* mRNA was induced upon senescence. Also, its induction closely paralleled the melatonin production in rice leaves. Also, rice ASMT mRNA was shown to be induced in abscisic acid (ABA), salt, and copper treatments, which suggested the possible involvement of melatonin in oxidative stress [27]. In 2013, Park et al. further determined that the over-expression of rice ASMT1, ASMT2, and ASMT3 independently enhanced enzyme activity [28]. Moreover, ASMT1 and ASMT2 transcripts were highly expressed in the stems and flowers. However, ASMT3 was barely detectable in any of the plant organs. All three ASMT mRNAs were simultaneously induced in the treatments with abscisic and methyl jasmonic acids [28]. In tomato plants, the transgenic plants’ over-expression of homologous sheep *oHIOMT* genes had higher melatonin levels, as well as enhanced drought tolerance, which provided new proof for the important role played by ASMT in plant melatonin synthesis [29]. Therefore, the accumulated evidence has demonstrated that ASMT is a very important enzyme for the biosynthesis of melatonin.

The tomato, as a model plant, has become an excellent material for the research interpretation of the various life activities of plants. Previously, according to an enzyme-linked immunosorbent assay, Okazaki et al. detected melatonin in the roots, stems, leaves, flowers, fruits, seedlings, and seeds, which ranged from 1.5 to 66.6 ng/g of the tomato plants’ fresh weight [2]. Sun et al. reported that an exogenous melatonin treatment significantly promoted ripening and improved tomato fruit quality post-harvest [30]. Arnao and Hernández-Ruiz reported that tomato plants under variable conditions exhibited a higher melatonin content [31]. Under abiotic stress, melatonin can enhance thermotolerance and cadmium phytotoxicity [32,33]. In addition, Li et al. reported that melatonin mediated selenium-induced tolerance to cadmium stress in tomato plants [34]. Subsequently, researchers found that HsfA1 not only induces drought tolerance but only upregulates melatonin biosynthesis to confer cadmium tolerance to tomato plants [35,36]. Overall, melatonin plays a very important role in regulating the growth and development, as well as controlling the environmental adaptation of tomato plants.

In this study, the *ASMT* gene family in tomato plants was characterized by the integration of structural features, phylogenetic relationships, chromosome localization, and the expression patterns in various tissues, as well as the responses to biotic stresses. This study’s in silico search identified a total of 14 putative *ASMT* genes in the *S. lycopersicum* genome. Evolutionary analysis revealed that tandem duplication events largely contributed to the expansion of the *SlASMT* gene family. A detailed expression analysis of the *SlASMT* genes, based on RNA-seq and qRT-PCR, showed distinct patterns of expression in the different organs and identified a number of *SlASMT* genes that were regulated by multiple pathogen infections. This study not only contributes to the understanding of the evolutionary patterns of the *SlASMT* genes in plants but also lays a foundation for deciphering the important functions of *SlASMT* in regulating melatonin biosynthesis in tomato plants.

## 2. Results

### 2.1. Identification of SlASMT Genes in the Tomato Genome

In previous research, three rice ASMT genes (*ASMT1*, *ASMT2* and *ASMT3*) were identified with different functions [27,28], leading to the hypothesis that plant species may contain a family of *ASMT* genes. In this current study, the BlastP tool was used to search the tomato genome database of the Sol Genomics Network (SGN, http://solgenomics.net/) using an amino acid sequence of the *O*-methyltransferase conserved domain as the query. Based on previous reports [26,37], we selected tomato ASMT homologs with more than 31% amino acid identities since the rice ASMT gene (a query) has about 31% aa identity to tomato caffeic acid *O*-methyltransferase (COMT). A total of fourteen non-redundant *SlASMT* genes candidates were identified (*SlASMT01 to SlASMT14*). Each of the three *SlASMT* genes (*SlASMT06*, *SlASMT09*, and *SlASMT13*) was missing significant portions of their coding regions. Therefore, it was most likely encoded truncated protein, or possibly pseudogenes. These three members were subsequently excluded from the phylogenetic and expression analysis. The characteristics of these *SlASMT* genes from the tomato plants were illustrated (Table 1), including the gene name, ID, and location, along with the number of exons, protein size, molecular weight (MW), and isoelectric point (p*I*).

### 2.2. Analysis of the SlASMT Gene Phylogeny and Exon-Intron Structure

**Figure 1:**
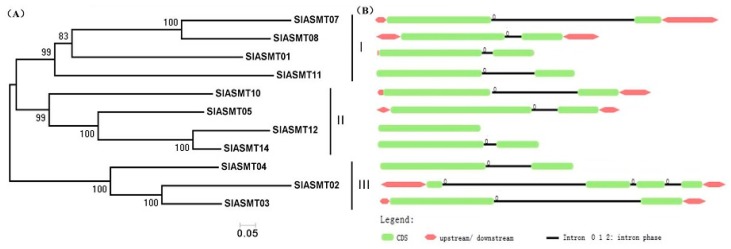
Phylogenetic analysis and intron/exon configurations of *SlASMT* genes in tomato plants. (**A**) Phylogenetic tree of the *SlASMT* genes was constructed using MEGA 5.0; (**B**) The introns and exons were drawn to scale with the full encoding regions of their respective genes. The boxes indicate the exons, and lines indicate the introns: 0 = intron phrase 0; 1 = intron phrase 1; 2 = intron phrase 2.

A Neighbor-Joining phylogenetic tree with 18*SlASMT* genes was constructed for the purpose of exploring the phylogenetic relationship of the *SlASMTs* in tomato plants using the MEGA5.0 program (Figure 1A). According to the phylogenetic tree topology, these genes could be divided into three groups (including Groups I to III). In order to examine the structural features of the *SlASMT* genes, the exon/intron configurations of *SlASMT* genes in the tomato plants were compared. The view of exon/intron structures was obtained using an online Gene Structure Display Server (GSDS: http://gsds.cbi.pku.edu.cn), with both coding sequences (CDS) and genomic sequences. Figure 1B provides a detailed illustration of the relative length of the introns and the conservation of the corresponding exon sequences within each of the *SlASMTs.* The results confirmed that various numbers of introns were found, and each of the *SlASMTs* contained fewer introns (0–3). In Group I, only one intron was observed in each member. The members of Group II included two introns, with the exception of the *SlASMT12*, which had no introns. In Group III, three introns were found in *SlASMT02*, and the remaining *SlASMT03* and *SlASMT04* had two introns. The results revealed relatively few introns in the process of the evolution of the tomato plants.

### 2.3. Genomic Distribution and Gene Duplication

**Figure 2:**
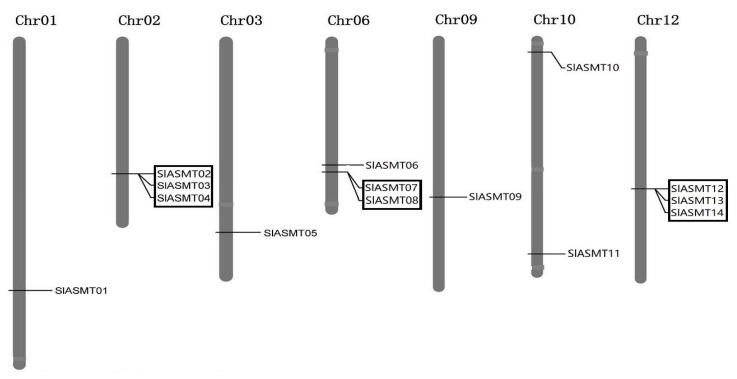
Position of *SlASMT* genes on the tomato chromosomes. The chromosome numbers are indicated at the top end of the chromosome. The tandem duplicated gene clusters are indicated by the black boxes.

In this study, chromosomal mapping was performed to examine the genome distribution of the *SlASMT* genes in the tomato plants. As the results indicated, multiple genes were located on seven of the twelve chromosomes and exhibited uneven distributions (Figure 2). A maximum number of three genes was mapped on chromosomes 02, 06 and 12, while only one gene was located on chromosomes 01, 03 and 09. In contrast, chromosomes 10 contained two members (*SlASMT10* and *SlASMT11*).

**Figure 3:**
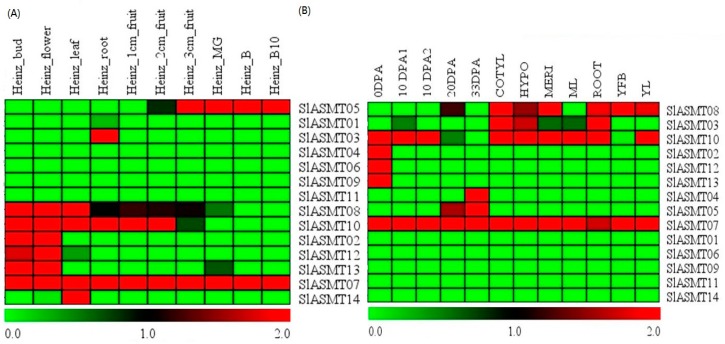
Expression profiles of *SlASMT* genes based on RNA-Seq in tomato plants. (**A**) Cultivated tomato (*S. lycopersicum*); (**B**) Wild tomato (*S. pinpinellifolium*); all RNA-Seq datasets were obtained from the Tomato Functional Genomics Database (http://ted.bti.cornell.edu/), and a detailed description of the samples is available from the Tomato Functional Genomics Database. Then, the log2-transformed RPKM values were used to obtain a heatmap using Multi-Experiment Viewer software. The blocks with colors indicate low (green) or high (red) transcript accumulation, relative to the respective control. MG is the mature green fruit; B represents the breaker stages (early ripening); and B10 represents the 10-day post breaker stages (red ripe).

It is well known that tandem and segmental duplications play roles in gene family expansion during plant evolution [38,39]. In this study, 10 (41.7%) of the *SlASMT* genes were clustered into three groups (A, B and C), and seemed to be produced from tandem duplications (Figure 3; Table 2). These three groups were juxtaposed, with no intervening gene (Table 2). The distance between these genes ranged from 0.128 kb to 139.18 kb (Table 2), and the overall similarity of the encoding sequences of these genes ranged from 38.2% to 60.2%. There were two genes in tandem in group B while groups A and C contained three genes each. However, no evidence of segmental genome duplications was identified for these *SlASMT* genes in the tomato plants. Therefore, these results suggested that tandem duplication events largely contributed to the expansion of the *SlASMT* gene family in the tomato plants.

### 2.4. SlASMTs Genes Have Specific Expression Patterns in Different Tomato Genotypes

All of the available RNA-Seq data in the Tomato Functional Genomics Database (http://ted.bti.cornell.edu/) were downloaded in order to decipher the expression pattern of the *SlASMT* genes among the various tissues of the tomato plants. The normalized gene expression values were estimated by the reads per kilo bases per million reads mapped (RPKM). Subsequently, the log2-transformed RPKM values were used to draw heatmaps using Mev4.9 software, and the results are shown in Figure 3. In this study, an in silico expression analysis was performed on various tissues in *S. lycopersicum*. As shown in Figure 3A, the results revealed that 9 of the 14 genes were expressed in all of the tested tissues. The transcripts of *SlASMT07* consistently appeared in all of the various tissues, while two of the genes (*SlASMT03*, and *SlASMT14*) showed tissue-specific expression profiles. Three of the genes (*SlASMT02*, *SlASMT12* and *SlASMT13*) showed similar expression profiles and were expressed in the buds and flowers. The gene *SlASMT05* was expressed exclusively in fruit. Expression profiles of the *SlASMT08* and *SlASMT10* genes were similar and were found under multiple tested conditions, including the bud, flower, leaf, root, 1 cm-fruit, 2 cm-fruit and 3 cm-fruit.

To further expand on the present knowledge of the expression profiles of the *SlASMT* genes in the different tissues, the expression patterns of the *SlASMT* genes in the different tissues of *S. Lycopersicum* and *S. pinpinellifolium* were compared (Figure 3B). The results showed that 9 of 14 genes were expressed in the tested tissues of *S. pinpinellifolium*. Seven of the genes (*SlASMT02*, *SlASMT07*, *SlASMT08*, *SlASMT05*, *SlASMT10*, *SlASMT12,* and *SlASMT13*) showed similar expression profiles in the different tissues of *S. lycopersicum* and *S. pinpinellifolium*. Two of the genes (*SlASMT03* and *SlASMT04*) showed different expression profiles in the different tissues of the two species.

### 2.5. SlASMT Genes are Regulated by Multiple Pathogens

**Figure 4:**
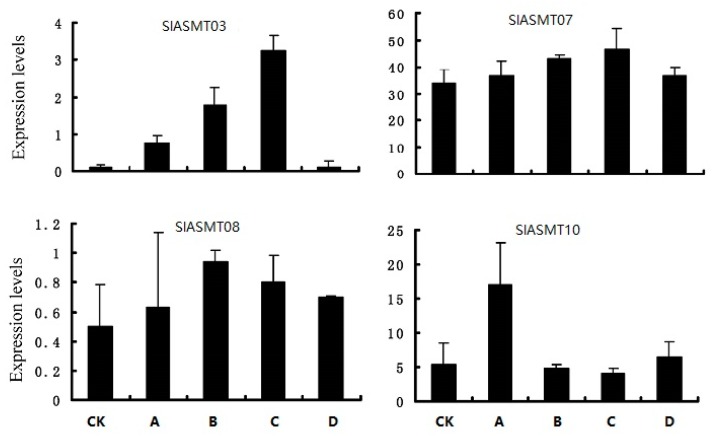
Expression patterns of *SlASMTs* in response to different pathogens. The axis of abscissas A, B, C and D represent *Pseudomonas syringae* tomato DC3000, *Pseudomonas fluorescens*, *Pseudomonas putida* and *Agrobacterium tumefaciens*.

Subsequently, the expression patterns of the *SlASMTs* were further analyzed in response to multiple pathogens, including *Pseudomonas syringae* tomato DC3000, *Pseudomonas fluorescens*, *Pseudomonas putida,* and *Agrobacterium tumefaciens*. The results showed that the expression abundances of 10 of the 14 genes were not detected under these biotic stresses. However, the remaining 4 genes were induced by these biotic stress factors (Figure 4). The *SlASMT03* and *SlASMT07* genes showed similar expression profiles and were up-regulated by the three pathogens, DC3000, *Pseudomonas fluorescens*, and *Pseudomonas putida*. The *SlASMT08* gene was weakly non-regulated under all of the tested biotic stress conditions, while *SlASMT10* genes were only up-regulated by *Pseudomonas fluorescens*, not *Pseudomonas syringae* tomato DC3000, *Pseudomonas putida,* or *Agrobacterium tumefaciens*.

### 2.6. Expression Divergence of the Tandem Duplicated SlASMT Genes in Tomato Plants

**Figure 5:**
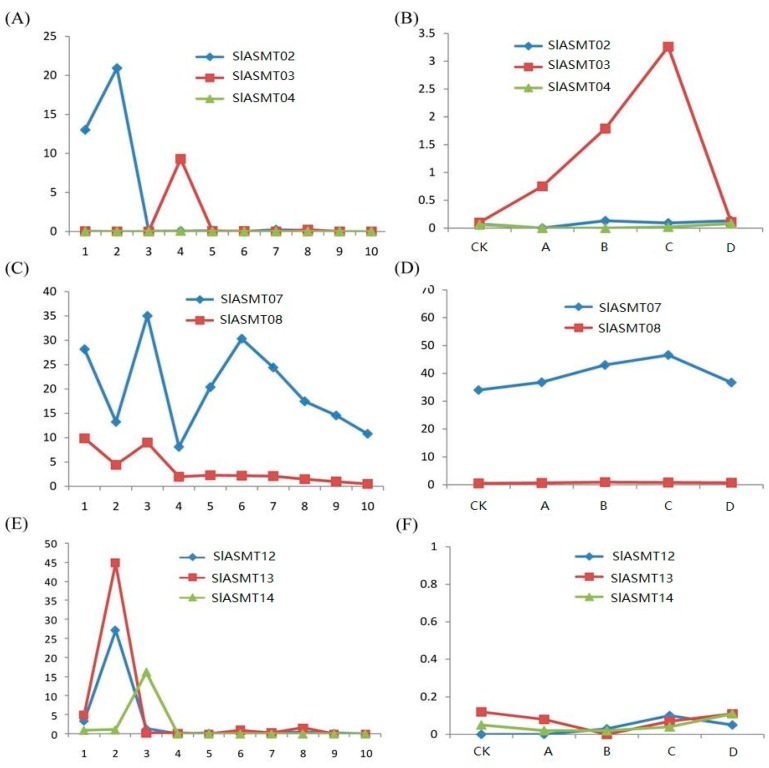
Expression patterns of *SlASMT* genes presented as tandem duplicates. The *X*-axis represents the developmental stages as shown in supplemental Table S2. The *Y*-axis represents the raw expression values obtained from RNA-seq.

In this study, the expression patterns of the tandem duplicated *SlASMT* genes were examined. The RPKM values were available for three groups in the RNA-seq data under the different conditions in this study. Genes in the first group (*SlASMT02*, *SlASMT03* and *SlASMT04*) showed divergent expression profiles. For example, the *SlASMT04* gene was not expressed at significant levels in all tested tissues (Figure 5A), which indicated that pseudo-functionalization after duplication could occur. Of these three genes, only the *SlASMT03* gene was induced by multiple pathogens (Figure 5B). The *SlASMT07* and *SlASMT08* genes of the second group showed similar expression profiles in different tissues and development stages (Figure 5C), while only the former was found to be induced by multiple pathogens (Figure 5D). In the third group (*SlASMT12*, *SlASMT13* and *SlASMT14*), the *SlASMT12* and *SlASMT13* genes revealed similar expression profiles, while the *SlASMY14* gene was expressed in the leaves (Figure 5E). Moreover, these three genes were not induced by the four pathogens (Figure 5F).

### 2.7. RT-qPCR Analysis

**Figure 6:**
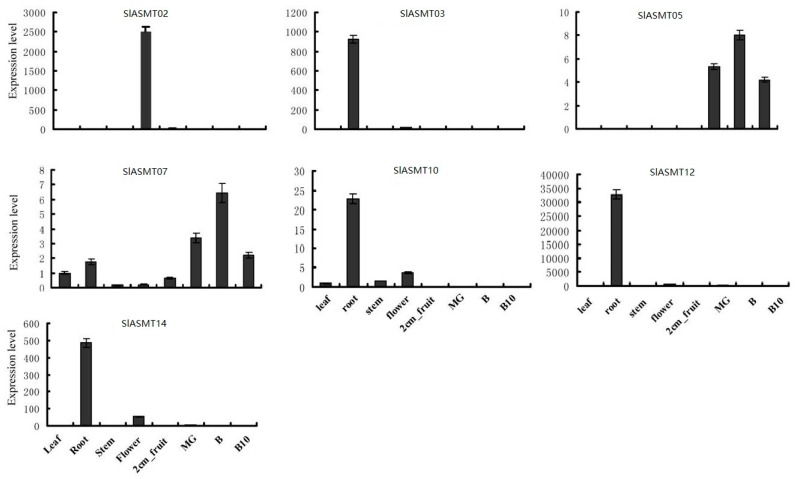
Expression patterns of *SlASMT* genes in different organs and developmental stages.

In order to confirm the results obtained by the RNA-Seq and to attempt to quantify the expression levels, qRT-PCR was performed, and the results were compared. In this study, the expressions of eleven *SlASMT* genes were analyzed for eight different tissues, including the roots, stems, leaves, flowers, immature green fruit, mature green fruit, breaker fruit, and 10-day fruit post breaker stages (red ripe). Among these eleven *SlASMT* genes, four (*SlASMT01*, *SlASMT04*, *SlASMT08* and *SlASMT11*) were not detected in any tissues. The remaining seven genes were expressed in at least one of the tested tissues (Figure 6). However, the expression levels of these genes displayed obvious differences. Three of the genes (*SlASMT02*, *SlASMT03* and *SlASMT12*) showed tissue-specific expression patterns. Of these seven *SlASMTs*, five were expressed in the roots, with the exception of the *SlASMT02* gene, which was expressed in the flowers. *SlASMT07* was ubiquitously expressed at similar levels in most of the tested organs. Additionally, the *SlASMT14* gene was expressed in the leaves based on the RNA-seq method. However, in this study, it was only detected in the roots and flowers when using qRT-PCR. Overall, these results were consistent with the expression of the *SlASMT* genes obtained by the RNA-Seq data.

## 3. Discussion

In previous studies, acetylserotonin-*O*-methyltransferase from rice, the last enzyme involved in melatonin biosynthesis, was cloned. It possesses partial homologies to other plant *O*-methyltransferases [28] and is localized in the cytosol [40]. Subsequently, two different genes (*ASMT2* and *ASMT3*), which were expressed at different levels, have been also reported in rice [41]. Given the multiple number of putative ASMT gene members identified in rice, it is possible that the ASMT gene family may exist in different plant genomes. Therefore, in this present study, in silico identification of the ASMT genes was performed by searching the *S. lycopersicum* genomic database (http://solgenomics.net/). The results showed a total of fourteen candidate non-redundant *SlASMT* genes, which indicated that the tomato genome encoded a family of *SlASMT* genes. Surprisingly, a large number of the pseudogenes (3/14 = 20%) was found in the tomato genome. The presence of such a high proportion of pseudogenes indicated that these genes could potentially play a role in the course of the plants’ evolution.

It is well known that gene duplications (tandem and segmental duplications) are one of the primary driving forces in the evolution of gene families with variations in size and distribution [38,39]. In tomato plants, the *SlASMT* gene family comprises a diverse family of proteins (Table 1). A total of 8 duplicated *SlASMT* genes were found to be present in the tomato chromosomes (Figure 2), and these were only involved in tandem duplications. Therefore, the gene duplication and subsequent expansion of the *SlASMT* genes seemed to have occurred throughout evolution. However, segmental duplication events were not observed in these genes. Therefore, in the present study, tandem duplication events have been determined to contribute significantly to the evolution of the *SlASMT* genes in tomato plants.

In addition, it was also found that the expression pattern of the tandem duplicated genes was highly variable within the three groups (Figure 5). For example, the sequence comparison between the *SlASMT02* and *SlASMT03* in the first group revealed a reduced level of homology at the amino acid level (56.27%), indicating that these genes might have undergone significant diversification after duplication, resulting in neo-functionalization. Also, in regards to the *SlASMT04* gene in the first group of the tandem duplicated genes, it did not show expression at significant levels in any of the tested tissues, which indicated that a pseudo-functionalization could have occurred after the duplication. This result may have been due to the fact that the genes with low levels of expression slowly lose their functions during the course of the evolutionary process. Therefore, the functional divergence of the *SlASMTs* occurred during the course of the evolution.

In order to investigate the possible functional differences of the *SlASMT* genes, their expression patterns were analyzed based on RNA-Seq and qRT-PCR technology. The results demonstrated different types of expression patterns among these *SlASMTs* (Figure 3 and Figure 6). Three of these genes (*SlASMT08* and *SlASMT10)* were expressed in the buds, flowers, leaves, roots, and non-mature fruit, which suggested an important role in the vegetative growth, as well as the early stages of reproductive growth. The expression of seven of the *SlASMT* genes showed tissue-specific patterns, including flowers and buds (*SlASMT02*, *SlASMT12* and *SlASMT13*), leaves (*SlASMT14*), and roots (*SlASMT03*). These results indicated a vital role in the growth and development of the flowers, leaves, and roots, respectively. In addition, it was also found that the *SlASMT05* gene was expressed in the mature fruit, which indicated that it could potentially play vital roles in the vegetative growth, as well as the latter stages of the fruit development. Finally, the expression of the *SlASMT07* gene was detected in all of the tested tissues and was involved in all growth and development stages of the tomato plants. In regards to the remaining five genes, their expressions were not detected in any of the tissues. These results suggested that the expression levels of these genes were too low to be detected in the tested tissues, or that they were not expressed to any significant degree.

The expression patterns of the *SlASMTs* in response to multiple pathogens were further analyzed, including *Pseudomonas syringae* tomato DC3000, *Pseudomonas fluorescens*, *Pseudomonas putida,* and *Agrobacterium tumefaciens*. The results showed that the expression of 10 of 14 genes was not detected under these biotic stresses. However, the remaining 4 genes were induced by these biotic stress factors (Figure 4). The *SlASMT03* and *SlASMT07* genes showed similar expression profiles and were up-regulated by three pathogens (*Pseudomonas syringae* tomato DC3000, *Pseudomonas fluorescens*, and *Pseudomonas putida)*. *SlASMT08* was weakly non-regulated under all of the tested biotic stress conditions. In addition, the *SlASMT16* genes were only up-regulated by *Pseudomonas fluorescens*. All the results suggested that these four genes are involved in tomato plant responses to biotic stresses.

## 4. Materials and Methods

### 4.1. Retrieval and Identification of the SlASMT Gene Family

In the present study, two methods were used to identify the potential AMST homologs in the tomato genome. First, a TBLASTN search was performed using the protein sequences of the ASMT genes from rice [27,28] as the query against the SGN tomato genome database (http://solgenomics.net/). Then, a HMM profile of the ASMT conserved domain (Pfam: PF00282) was downloaded from the Pfam protein family database (http://pfam.sanger.ac.uk/). For the purpose of identifying the *SlAMST* genes, BlastP was used in the *S. lycopersicum* genome. The default parameters were employed. All of the non-redundant gene sequences were searched against the tomato genome data of the SGN (http://solgenomics.net/). The *e*-value used was 1 × 10^−5^. The conserved NBS domain of these predicted NBS-encoding proteins was determined by Pfam version 22.0 (http://pfam.janelia.org). Subsequently, the molecular weights and the isoelectric point of the *SlAMSTs* deduced proteins were predicted using the online tool ExPASy (http://web.expasy.org/protparam/).

### 4.2. Mapping of the SlASMT Genes on the S. lycopersicum Genome and the Determination of the Exon-Intron Structure

In order to determine the location of the *SlASMT* genes on the 12 tomato chromosomes, the data concerning the gene positions were extracted from the tomato Genome Sequence ITAG Release 2.4 in the SGN (http://solgenomics.net). The software MapDraw 2.2 was used to visualize the locations of the *SlASMT* genes on the tomato chromosomes. The tandem duplicated genes were defined as being closely related genes on a single chromosome, with a maximum of ten intervening genes [42]. The segmentally duplicated genes were detected by a CoGeSynMap program (http://genomevolution.org/CoGe/SynMap.pl). Additionally, in order to further investigate the structural characteristics of the *SlAMSTs* genes family, the genome sequence, coding sequence (CDS), and protein sequence of the homologous genes of the *SlAMSTs* in the tomato plants were downloaded from the SGN (http://solgenomics.net). A schematic diagram of the intron-exon structure of the *SlAMSTs* genes was depicted by the online tool Gene Structure Display Sever (version 2.0) (http://gsds.cbi.pku.edu.cn/).

### 4.3. Phylogenetic Tree Constructions

In order to determine the phylogenetic relationships of the *SlAMST* genes in tomato plants, multiple sequence alignments of the SlAMST protein sequences were performed using Clustal software (version 2.0) [43] and were then encoded by a BioEdit Sequence Alignment Editor [44]. Then, a phylogenetic tree was constructed using MEGA 5.0 software and a Neighbor-Joining method [45,46]. Bootstrap analysis was performed using 1000 resampling replications, and the branch lengths were assigned through the pair-wise calculations of the genetic distances. The missing data were treated as pair-wise deletions of the gaps.

### 4.4. Expression Analysis of the SlASMT Genes Based on RNA-seq

The widespread application of RNA-seq data was convenient for detecting the differential expression of the genes [47]. In this study, for the purpose of deciphering the expression pattern of the *SlAMST* gene family in the tomato plants’ various tissues, as well as the responses to biotic stresses, all available transcriptome data of the *SlAMSTs* genes were obtained from the Tomato Functional Genomics Database (http://ted.bti.cornell.edu/). This was followed by the obtained expression data being submitted to a Multiple Experiment Viewer (Verision Mev 4.9) software program with a log_2_ transformation in order to generate a heat map [48]. The obtained data were hierarchically clustered, based on Pearson’s correlation distance with average linkage, and a cluster analysis was performed on the rows of the expression values.

### 4.5. RT-qPCR Analysis

At this point, to further verify the expression pattern of the *SlASMTs*, eight tissues samples were obtained. These tissue samples included roots, stems, leaves, flowers, immature green fruit, mature green fruit, breaker fruit, and red fruit, obtained from *S. lycopersicum* L. var zhefen702, which had been grown in a controlled environment chamber at the Zhejiang Academy of Agricultural Sciences. Then, the total RNA was extracted, and the first-strand cDNA was synthesized using a RNA simple Total RNA Kit (Tiangen, Beijing, China), and a TIANScript cDNA Synthesize Kit (Tiangen Biotech, Beijing, China), respectively, both in accordance with the manufacturer’s instructions. Supplemental Table S1 lists the gene-specific primers of the *SlASMTs* for the qRT-PCR. The real-time PCR reactions were carried out in a total volume of 20 μL, containing 10 μL of SuperMix, 0.4 μL of each primer, 1 μL of template (10× the diluted cDNA from the samples), and 7.8 μL of sterile distilled water. The thermal conditions were as follows: 95 °C for 30 s; followed by 40 cycles at 95 °C for 5 s; 55 °C for 15 s; and 72 °C for 10 s. The relative gene expression values were calculated using the 2^−ΔΔ*C*t^ method. GAPDH was used as a reference gene for the expression analysis of the *SlASMT* genes in the tomato plants [49], and three independent replicates were then performed.

## 5. Conclusions

Here, we tried to characterize the *ASMT* gene family in tomato plants by the integration of structural features, phylogenetic relationships, chromosome localization, and the expression patterns in various tissues, as well as the responses to biotic stresses. In summary, this study identified fourteen members of the *SlASMT* gene family in tomato plants, and elaborated their phylogenetic relationships and evolutionary modes. Further analysis determined that several *SlASMT* genes displayed tissue-specific expression profiles. The results of this study lay the foundation for the deciphering of the function of the *SlASMT* family members in tomato plants.

## Figures and Tables

**Table 1:** Tomato ASMT gene family members.

Name	Locus Gene	Chromosome Location	No. of Exons ^a^	ORF Length ^b^ (bp)	Deduced Polypeptide		
					Length ^c^ (aa)	Mw (kDa) ^d^	p*I* ^e^
SlASMT01	Solyc01g068550	Chr1: 70160716..70161864	1	1059	352	39.06	5.13
SlASMT02	Solyc02g077510	Chr2: 36987681..36990211	3	816	271	29.99	4.79
SlASMT03	Solyc02g077520	Chr2: 36995587..36997979	1	1074	357	40.20	5.46
SlASMT04	Solyc02g077530	Chr2: 37009970..37011385	1	1083	360	40.71	5.56
SlASMT05	Solyc03g097700	Chr3: 53478085..53476300	1	1338	445	50.00	6.73
SlASMT06	Solyc06g060200	Chr6: 34560869..34562595	2	381	126	14.19	9.02
SlASMT07	Solyc06g064500	Chr6: 36552243..36549724	1	1008	335	35.52	5.12
SlASMT08	Solyc06g064510	Chr6: 36561810..36560172	1	1068	355	39.46	5.07
SlASMT09	Solyc09g056230	Chr9: 43236403..43235763	2	417	138	15.19	4.33
SlASMT10	Solyc10g008120	Chr10: 2265625-2267632	1	1089	362	40.29	5.61
SlASMT11	Solyc10g079540	Chr10: 60389269..60387816	1	1068	355	40.30	4.73
SlASMT12	Solyc12g041940	Chr12: 41005906..41006655	0	750	249	27.98	5.69
SlASMT13	Solyc12g041950	Chr12: 41006783..41007094	0	312	103	11.79	5.36
SlASMT14	Solyc12g041960	Chr12: 41147454..41146274	1	1089	362	40.84	6.27

^a^ No. of exons with coding domain sequence; ^b^ Length of open reading frame; ^c^ Length (no. of amino acid) of the deduced polypeptide; ^d^ Molecular weight of the deduced polypeptide in Daltons; ^e^ Isoelectric point of the deduced polypeptide.

**Table 2:** Tandem duplicated *SlASMT* genes.

Group	Gene	Duplicate	%Homology ^a^	Genes Intervening	Distance (bp)
A	SlASMT03	SlASMT04	56.27	0	5376
A	SlASMT04	SlASMT05	74.31	0	11,911
B	SlASMT07	SlASMT08	69.27	0	7929
C	SlASMT12	SlASMT13	10.20	0	128
C	SlASMT13	SlASMT14	24.59	0	13,9180

^a^ Homology between proteins encoded by tandem duplicated genes.

## Supplementary Materials

The following are available online, Table S1: Primers used to detect the expression of tomato *SlASMT* genes in this study. Table S2: The developmental stages and leaves infected by different pathogens are represented on the *X*-axis.
