# Supplementary material for: Genomic Analysis of the ASMT Gene Family in Solanum lycopersicum
Source: Molecules. 2017 Nov 16;22(11):1984. doi: 10.3390/molecules22111984 (PMC6150316; doi:10.3390/molecules22111984)
Supplement: Supplementary file 1 [file molecules-22-01984-s001.pdf]

Table S1 Primers used to detect expression of tomato *AlASMT* genes in this study

Name	Locus gene	Forward primer (5' to 3')	Reverse primer (5' to 3')
SlASMT01	Solyc01g068550	GGAATCCCTGATGTTCTG	GAAACTGCATTAAAATTGGT
SlASMT02	Solyc02g077510	AATTTCATTGAGACGAGCCA	TGAAGACATAACGGACATGGA
SlASMT03	Solyc02g077520	TGTTTGAAGCTCAGGCTCAT	GCTGAGACAGAGTCATTGGC
SlASMT04	Solyc02g077530	TGTGCCATTCAGTTAGGCAT	AGAATTGAGCGTGCACCA
SlASMT05	Solyc03g097700	TTAGGAAGATTAGGTAAA	GAAATGTTATCTTGGTAT
SlASMT07	Solyc06g064500	GAAGCTGAAGCTCAAAGTTGG	TCAGAAAGAGACATGATCGGA
SlASMT08	Solyc06g064510	TTTAATGGGATAGAACCA	GATCGAATAAGATTTGAATG
SlASMT10	Solyc10g008120	CCATGTCACTCAAATCAGCA	AGGGCATTGGCTAATTCATC
SlASMT11	Solyc10g079540	TCACCAAACATGACAAACCA	TATTCGTGTAAGGCGGTGAA
SlASMT12	Solyc12g041940	ACATTGCCCAATGACAACAT	CGGCCATGAGAGTGGATATT
SlASMT14	Solyc12g041960	GGCATTGCCTAATGACAACA	TGCACATTTGAGTGACATGG

Table S2 The developmental stages and leaf infected by different pathogens are represented in *X*-axis

Sample	Developmental stages analyzed
1	Hernz_bud
2	Hernz_flower
3	Hernz_leaf
4	Hernz_root
5	Hernz_1cm_fruit
6	Hernz_2cm_fruit
7	Hernz_3cm_fruit
8	Hernz_MG (mature green fruit)
9	Hernz_B (breaker stages fruit)
10	Hernz_B10 (10 days post breaker stages fruit)
CK	leaf
A	*Pseudomonas syringae* tomato DC3000
B	*Pseudomonas fluorescens*
C	*Pseudomonas putida*
D	*Agrobacterium tumefaciens*
